# Role of diffusion MRI in characterizing benign and malignant breast lesions

**DOI:** 10.4103/0971-3026.57209

**Published:** 2009-11

**Authors:** Lalitha Palle, Balaji Reddy

**Affiliations:** Department of Radiology, Focus Diagnostics, Hyderabad, Andhra Pradesh, India

**Keywords:** Apparent diffusion coefficient, diffusion, MRI

## Abstract

**Aims:**

The aim of this study was to evaluate the role of MRI based diffusion-weighted imaging (DWI) and the apparent diffusion coefficient (ADC) for characterizing breast lesions in Indian patients.

**Materials and Methods:**

This prospective analysis was performed between October 2006 and June 2008. It includes 200 patients between the ages of 16 and 80 years with solid breast lesions greater than 1 cm in diameter. Of these 200 patients, 80 underwent breast MRI with contrast and DWI. One hundred and twenty patients had only DWI as they had come only for sonomammography. A total of 280 lesions were detected. ADC values were calculated for all the lesions and the highest and lowest values of ADC for benign and malignant lesions were identified. Finally, we compared our findings with those of previous studies.

**Results:**

Two hundred and eight lesions were categorized as benign and 72 lesions were categorized as malignant based on the ADC values. Based on previous data, lesions with ADC values from 1.3 to 1.5 mm^2^/s were considered benign where as lesions with ADC values ranging between 0.85 and 1.1 mm^2^/s were considered malignant. Two lesions whose ADC values were in the benign range were proven to be malignant tumors after surgery. This method of using ADC values for the detection of malignant lesions showed a sensitivity of 97.22% and a specificity of 100%. The positive predictive value was 100%.

**Conclusion:**

DWI is a useful technique for characterizing breast tumors, especially for lesions that cannot be adequately characterized by ultrasonography and routine magnetic resonance imaging.

## Introduction

Diffusion-weighted MRI imaging (DWI) is widely used, especially for the evaluation of acute cerebral infarction. It has recently been used to evaluate other organs such as the liver, prostate, ovaries, pancreas, and the breast.[1] Some studies have found that DWI has the ability to differentiate benign from malignant lesions.[1] Our study was also performed to evaluate the role of DWI in differentiating benign from malignant breast lesions.

## Materials and Methods

This study includes a total of 200 patients (199 female patients and one male patient) between the ages of 16 and 80 years who had come for imaging between October 2006 and June 2008. Those patients who had come for breast MRI examinations and were detected to have lesions greater than 1 cm in size were included in the study and DWI was performed after obtaining prior consent. Patients who were referred for mammography and sonomammography and who were detected to have solid lesions greater than 1 cm were also included in this study after obtaining prior consent; diffusion-weighted sequences were performed for these patients only to characterize the lesions detected on mammography and sonomammography. Purely cystic lesions, even those that were later proven to be malignant, show no significant restriction within the lesion and therefore were not included in this study. There were two such lesions. Lesions greater than 1 cm in size were selected for this study because only such lesions are clearly identifiable on the DWI images for the placement of a region of interest (ROI) entirely within the lesion.

## MRI protocol

The images were acquired with a 1.5-T scanner (Magnetom Avanto, Siemens, Germany). The patients were placed in a prone position in a breast coil and MRI was performed using the following sequences: T2W axial sequence (TR/TE: 4500/110), number of excitation (NEX): 1, slice thickness: 3 mm, and field of view (FOV): 350 mm; T1W axial (TR/TE: 450 /13), NEX: 1, slice thickness: 3 mm, and FOV: 350 mm; STIR axial (TR/TE: 9500/100), NEX: 1, slice thickness: 3 mm, and FOV: 350 mm. Five continuous dynamic contrast-enhanced FLASH 3D acquisitions were performed (TR/TE: 4.4/1.6, flip angle: 12°, and slice thickness: 1 mm). The time taken for each acquisition was around 1.2 min. A 3D FLASH sagittal acquisition was also performed (TR/TE: 26/6.4 and flip angle: 30°).

Axial DWI with single-shot echo-planar imaging (EPI) was performed at b values = 0, 500, and 1000, TR/TE: 1800/75, FOV: 350 mm, and slice thickness: 3 mm. Diffusion imaging was performed at b values of 0, 500, and 1000. The apparent diffusion coefficient (ADC) values were automatically calculated by placing the ROI well within the confines of the lesion. Fatty glandular parenchyma, which shows homogeneous signal intensity on the ADC map, was used as a reference. The ADC values were automatically measured by drawing ROIs. The size of the ROI was 0.03 cm^2^. The scanner software provides the mean value within the ROI, which equals the ADC value (multiplied by 10^−3^).

## Results

**Figure 1 (A,B):**
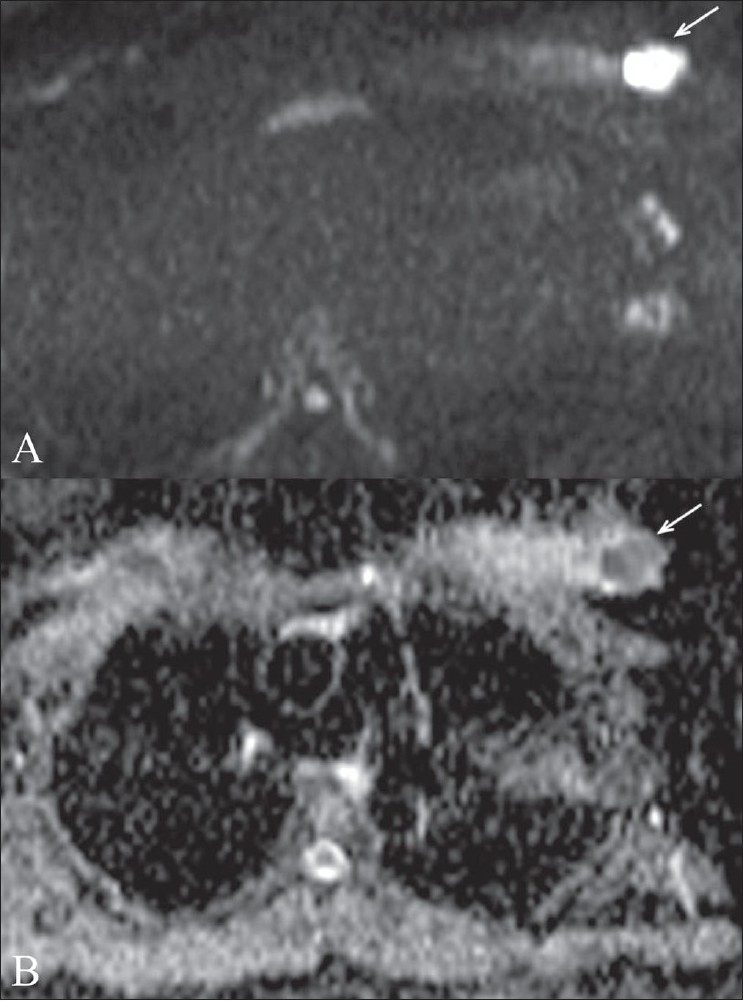
Invasive ductal carcinoma. Diffusion-weighted image (A) at a b value of 1000 shows a malignant mass (arrow). Apparent diffusion coefficient (ADC) mapping (B) reveals restricted diffusion in the mass (arrow). The ADC value was 0.95 mm^2^/s

**Figure 2 (A,B):**
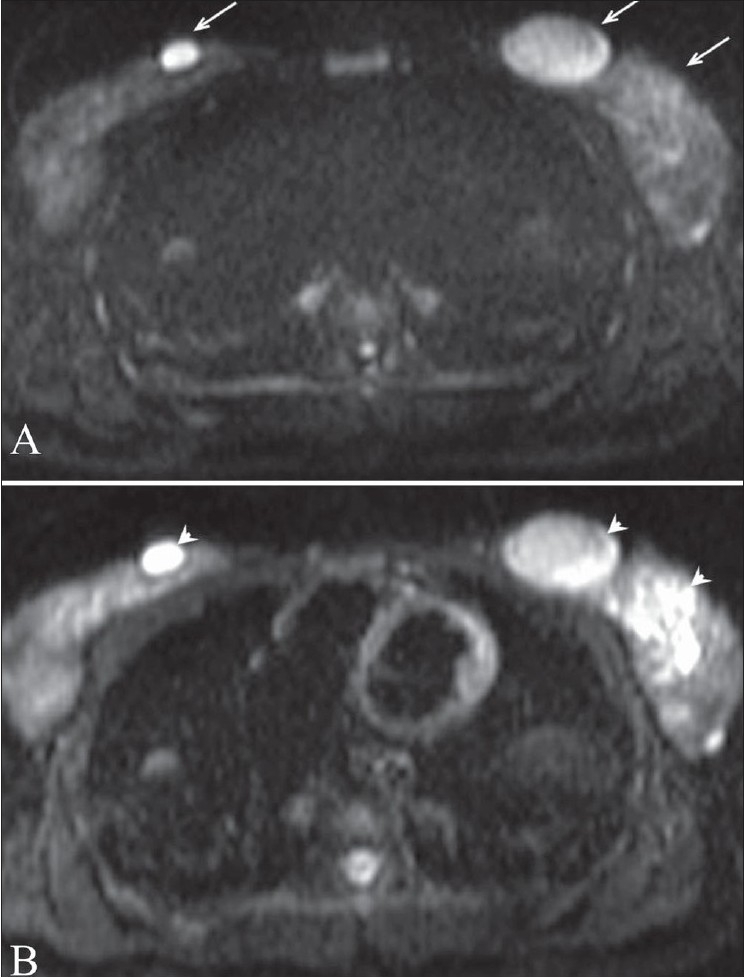
Fibroadenoma. Diffusion-weighted image (A) at a b value of 1000 shows bilateral breast fibroadenomas (arrows) Apparent diffusion coefficient (ADC) mapping (B) reveals no restricted diffusion in the lesions (arrowheads). ADC values are 1.3 and 1.34 mm^2^/s in the left and right breast lesions, respectively

**Figure 3:**
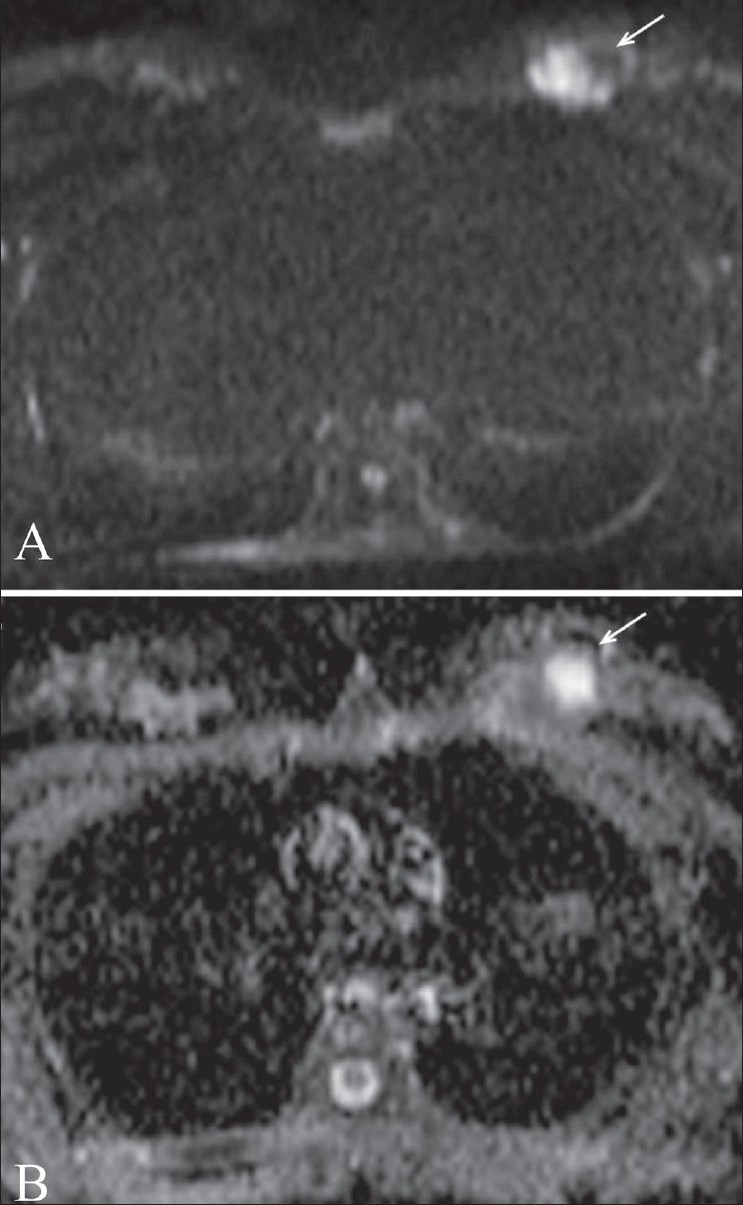
(A,B): False-negative result in a patient with invasive ductal carcinoma. Diffusion-weighted image (A) at a b value of 1000 shows a left breast mass (arrow) with solid and cystic components. This lesion was proven to be malignant after surgery. Apparent diffusion coefficient (ADC) mapping (B) reveals restricted diffusion (arrow) in the solid component of the mass. The region of interest containing the solid and cystic component reveals an ADC value of 1.5 mm^2^/s

Two hundred and eighty breast lesions were detected in our sample of 200 patients. Based on previous experience, lesions with ADC values in the range of 0.89 ± 0.18 × 10^−3^ mm^2^/s were called malignant [Figure 1] and those between 1.41 ± 0.56 × 10^−3^ mm^2^/s were called benign[2] [Figure 2]. For normal breast parenchyma, the ADC values were between 1.59 and 1.7 × 10^−3^ mm^2^/s. Of the 280 lesions, 208 lesions were categorized as benign and 72 lesions were categorized as malignant using these ADC values. Two lesions whose ADC values were in the benign range turned out to be malignant after surgery [Figure 3]. Both these lesions also showed type 2 curves on the dynamic contrast-enhanced study. According to this study, the sensitivity of ADC values for the detection of malignant lesions was 97.22% and the specificity was 100% and the positive predictive value was 100% and the negative predictive value was 99%.

## Discussion

DWI provides important biological information about the composition of tissues, their physical properties, their microstructure, and their architectural organization.[3] This information is available noninvasively and without contrast administration. DWI generates images that are based on the molecular motion of water, which is altered by disease.[4]

DWI is based on the principle of Fick's Law of concentration gradients and the Brownian movement of molecules. Malignant lesions, in general, have more tightly packed cells with a more compact architecture and, consequently, have lower ADC values as compared with benign lesions. There is inhibition of effective movement of water molecules and restricted diffusion in dense malignant lesions. The higher ADC values of cystic or necrotic areas reflect a lack of significant restriction of diffusion of water.[5] False-negative values can be obtained in cystic/necrotic malignancies.[5]

We have found that using cut-off ADC ranges of 1.3−1.5 × 10^−3^ mm^2^/s for benign lesions and 0.85–1.1 × 10^−3^ mm^2^/s for malignant lesions allows differentiation of benign from malignant lesions with a high sensitivity and specificity. They were two false negatives for malignancy. Both these lesions had small cystic components, which were included in the ROI, leading to high ADC values.

In the study conducted by Zhang *et al.* on 57 breast lesions, the threshold ADC value at b-1000 was 1.20 ± 0.25 × 10^−3^ mm^2^/s.[6] They found that ADC values of malignant lesions were statistically much lower than benign lesions and peritumoral tissues.[6] The usefulness of contrast MRI in detecting breast malignancy was studied by Drew *et al.* in 334 women.[7] The sensitivity and specificity of dynamic contrast MRI in their study were 100% and 86% respectively for detecting malignant lesions.[7] In a study performed by Yabuuchi *et al.* to assess the utility of a combination of dynamic contrast MRI and DWI in lesion characterization, the sensitivity was found to be 92% and the specificity was found to be 86% for differentiating benign from malignant lesions.[8]

The results in our study are better than other studies probably because all the lesions were above 1 cm and therefore the ROI was placed well within the lesion, reducing the possibility of false sampling from adjacent tissues. All our cases, except one, were completely solid lesions and hence there was no cystic component in the ROI, which would otherwise have altered the ADC values.

## Conclusion

Based on our preliminary data, we have found that DWI for breast lesions can differentiate benign from malignant lesions with a high sensitivity and specificity. The usefulness of this technique needs to be further evaluated with larger double-blind studies.
